# Surface Roughness and Color Stability of 3D-Printed Dental Resin Materials Under the Influence of Different Beverages

**DOI:** 10.3390/jfb17100530

**Published:** 2026-10-20

**Authors:** Walaa Magdy Ahmed, Amr Ahmed Azhari, Basem Nori Alturki, Abdullah Omar Batarfi, Loay Algizani, Mohamed Gebril, Yasser M. Merdad, Hanadi Yousif Marghalani, Ricardo Marins de Carvalho

**Affiliations:** 1 Dental Restorative Biomaterials, Biomechanics and Adhesion-Research Group (DRBBA-RG), Department of Restorative Dentistry, Faculty of Dentistry, King Abdulaziz University, Jeddah 21589, Saudi Arabia; aaaazhari@kau.edu.sa (A.A.A.); hmarghalani@kau.edu.sa (H.Y.M.); 2 Department of Prosthodontics, Makkah Health Cluster Healthcare, King Faisal Hospital, Makkah 24236, Saudi Arabia; balturki@moh.gov.sa; 3 Department of Prosthodontics, Taif Health Cluster, Taif Dental Specialized Center, Taif 26514, Saudi Arabia; aobatarfi@moh.gov.sa; 4 Oral and Maxillofacial Prosthodontics Department, King Abdulaziz University Dental Hospital, Jeddah 21589, Saudi Arabia; lalgizani@kau.edu.sa; 5 Department of Oral Health Sciences, Faculty of Dentistry, University of British Columbia, Vancouver, BC V6T 1Z3, Canada; 6 Department of Restorative Dentistry, Faculty of Dentistry, King Abdulaziz University, Jeddah 21589, Saudi Arabia; ymerdad@kau.edu.sa; 7 Department of Oral Biological and Medical Sciences, Faculty of Dentistry, University of British Columbia, Vancouver, BC V6T 1Z3, Canada; rickmc@dentistry.ubc.ca

**Keywords:** surface roughness, color stability, 3D-printed resin, subtractive manufacturing, additive manufacturing, provisional restorations

## Abstract

**Aim:** This study aimed to evaluate the effects of the material type and immersion solution on the color stability (ΔE) and surface roughness change (ΔRa) of provisional dental materials fabricated using subtractive milling or additive 3D printing techniques. **Materials and Methods:** Three provisional restorative materials were evaluated: one CAD/CAM-milled material (Vita CAD-Temp) and two 3D-printed materials (NextDent C&B MFH and Freeprint Crown [Detax]). The specimens were subjected to thermocycling and subsequently immersed for two weeks in coffee, hibiscus tea, Coca-Cola, or distilled water. Color change was evaluated using independent specimens (n = 10 per material × solution combination; N = 120), and ΔE was calculated from paired baseline and post-immersion CIELAB measurements. Surface roughness change was evaluated using a separate specimen set (n = 5 per material × solution combination; N = 60) and calculated as ΔRa = Ra_post-immersion−Ra_baseline. Factorial analyses were performed to assess the effects of the material, immersion solution, and their interaction, including multiplicity-adjusted post hoc comparisons where appropriate. **Results:** Color change was significantly affected by material type (*p* < 0.001), immersion solution (*p* < 0.001), and the material × solution interaction (*p* < 0.001). The interaction effect demonstrated that material performance depended strongly on the immersion solution. The highest mean ΔE was observed for NextDent exposed to hibiscus tea (8.96 ± 0.75), followed by Detax exposed to coffee (7.38 ± 1.69), whereas the lowest values were observed for milled material in distilled water (1.41 ± 0.45) and NextDent in cola (1.52 ± 1.00). For surface roughness, the immersion solution had a significant effect on ΔRa (*p* < 0.001), whereas the overall material effect (*p* = 0.136) and material × solution interaction (*p* = 0.706) were not statistically significant. Cola produced the greatest overall increase in surface roughness. **Conclusions:** The color stability of the tested provisional materials was dependent on the material type and immersion solution, with a pronounced interaction observed between these factors. The milled material generally demonstrated relatively low color changes across the tested conditions, while the responses of the 3D-printed materials varied substantially according to the immersion solution. In contrast, the change in surface roughness was influenced primarily by the immersion solution rather than material type. These findings indicate that the behaviors of provisional restorative materials should be interpreted according to the specific material–environment combination rather than the fabrication method alone.

## 1. Introduction

In the context of modern dental technology, color-matching between restorations and natural teeth is an important consideration [1]. Provisional restorations are integral to prosthetic treatments, providing protection to the teeth and surrounding periodontal tissues throughout the restorative process [2,3]. These restorations are essential to maintain phonetics, mastication, and esthetics while also ensuring tooth position stability, proper occlusion, comfort, and functionality [3] and offer an initial preview of treatment outcomes before finalization [4,5,6]. When choosing materials for provisional restorations, it is therefore essential that they fulfill biological, esthetic, and mechanical criteria. Furthermore, the following factors should be taken into consideration: the durability of the material, the heat generated during polymerization, the degree of shrinkage post-polymerization, the integrity of the margins, the protection of the dental pulp, and color retention [3,7]. Multi-Criteria Decision-Making (MCDM) methods have been widely used for the selection of an optimal material but are often computationally intensive [8].

While no singular material meets all these criteria, specific materials have proven effective in dental practice [9]. Depending on the complexity of the treatment, these restorations may be required for an extended period of time; thus, their structural stability, surface integrity, and esthetics need to be maintained. As alterations may cause significant esthetic problems, potentially leading to patient dissatisfaction and the need for material replacement, ensuring the color stability of the used material is essential [10,11,12,13]. Selecting an appropriate restorative material is essential for achieving successful results in areas where esthetics are of paramount importance, and the way these materials stain over time is a critical consideration [10,11,12,13]. Over time, provisional materials may become susceptible to degradation, plaque accumulation, gingival irritation, staining, discoloration, and eventual fracture due to daily wear and thermal stress in the oral environment [10,11,12,13]. Changes in the total color (ΔE) and surface roughness (ΔRa) of these materials have significant implications, especially in the esthetic zone [9].

The production of temporary restorations has progressed from traditional techniques using auto-polymerized resins to sophisticated methods incorporating computer-aided design (CAD) and computer-aided manufacturing (CAM), including subtractive milling and additive 3D printing [2,14]. At present, provisional restorative materials are commercially available as pre-polymerized milled blanks or as light-curable resins for additive manufacturing (3D printing). Milled provisional materials are typically PMMA- or bis-acrylic composite-based blanks that are industrially polymerized under controlled conditions, resulting in a more homogeneous, highly cross-linked structure. In contrast, 3D-printed provisional resins are photopolymerized methacrylate- or urethane-dimethacrylate-based systems, formulated with varying monomer chemistries, photoinitiators, and filler content depending on the manufacturer. These differences in the organic matrix and filler composition—rather than the fabrication method alone—can influence the water sorption properties, color stability, and surface characteristics of the final restoration [9].

CAD/CAM provisional materials offer advantages including reduced heat generation during fabrication and enhanced resistance to masticatory forces, leading to more durable results; however, they are typically more expensive [14,15].

Additive manufacturing (3D printing) has revolutionized the fabrication of dental parts due to its ability to create complex geometries with high precision while also significantly decreasing fabrication costs and processing times. The ability of these restorations to resist surface and color alterations is influenced by numerous factors, including the fabrication method, material type, surface texture, and oral hygiene [14,15]. Several 3D printing techniques are suitable for dental applications, including stereolithography (SLA), digital light processing (DLP), fused deposition modeling (FDM), selective laser sintering (SLS), and multi-jet printing (MJP). Each of these techniques offers distinct advantages, depending on the required resolution, material properties, and fabrication speed. SLA and DLP are widely utilized in dental settings due to their high accuracy and smooth surface finish, making them ideal for creating dental models, surgical guides, and crowns. FDM, while less precise, is cost-effective and suitable for prototyping. SLS and MJP enable the use of a broader range of materials, including biocompatible polymers and ceramics, which are critical for dental implants and prosthetics.

Although research has been conducted to evaluate the ΔE and Ra values of dental provisional restorative materials following exposure to various staining agents and mouth rinses [16,17], there are limited available studies addressing such changes in 3D-printed dental provisional restoratives after exposure to different immersion solutions.

Therefore, this study aimed to assess the impacts of various beverages and material types on the color stability (ΔE) and surface roughness (ΔRa) of provisional dental materials produced through milling or 3D printing. The two following null hypotheses were evaluated: (1) Color change is not considerably affected by the type of material, the immersion solution, and the interaction between the two. (2) There is no substantial influence of immersion solution, material type, and the interaction between them on the change in surface roughness.

## 2. Materials and Methods

This study evaluated the color stability (ΔE) and surface roughness (Ra) of three provisional dental materials in four different immersion solutions. The materials comprised the subtractive CAD/CAM composite Vita CAD-Temp (Vita Zahnfabrik, Bad Säckingenc, Germany) and two additive 3D-printed resins, Nextdent (Nextdent C&B MFH, 3D Systems, Soesterberg, The Netherlands) and Freeprint Crown (DETAX GmbH, Ettlingen, Germany). A1 shade was selected for the three materials. For the assessment, we used three different immersion solutions—namely, coffee, (Nescafe Classic, prepared with 2.8 g per 150 mL of boiling distilled water, pH 4.5), carbonated drink (Coca-Cola, undiluted, pH 2.5), and hibiscus tea (naturally dried, pH 6)—as well as distilled water as a control.

Vita CAD-Temp was prepared by grinding pre-polymerized blocks into cylinders using a grinding machine (buehler metaserv 250, lake bluff, IL, USA), then cutting them into discs (14 mm diameter, 1.1 mm thickness) with a water-cooled Isomet cutting machine, operating at 300 RPM under a 150 g load. Nextdent and Freeprint Detax Crown specimens were designed digitally to the same disc dimensions using the Lychee Slicer software 8.0.1 (Mango 3D, Bordeaux, France) and printed using an Asiga Max UV printer (Asiga, Alexandria, New South Wales, Australia) with a resolution of 20 μm, orienting samples at a 90° angle. After printing, the samples were cleaned with isopropyl alcohol (>99% purity), sonicated for 3 min, and cured under UV light at a wavelength of 385 nm for 20 min. All samples underwent a standardized polishing procedure using a Buehler Ecomet 250 machine (Buehler, lake Bluff, IL, USA) equipped with silicon carbide papers from 600 to 2500 grit, under water cooling at 300 RPM for 3 min per grit. After polishing, the disc specimens were subjected to ultrasonic cleaning for 10 min.

Forty specimens per solution group (coffee, hibiscus tea, carbonated drink, distilled water) were prepared and randomly divided, with each group containing ten samples per material (n = 10). The tests were conducted at a controlled temperature of 37 °C, simulating the oral environment. The specimens were immersed in static immersion media to maintain consistent exposure and reproducibility. The immersion media were refreshed every 48 h during the 2-week immersion period to avoid saturation. After the immersion period, the specimens were rinsed with distilled water to remove surface precipitants or debris.

Baseline measurements for color and surface roughness were captured. Then, all samples underwent thermocycling (TC), alternating between 5 and 55 °C, with an immersion time of 30 s at each temperature and 10 s at room temperature for transition, totaling 30,000 cycles over two weeks, simulating approximately two years of oral use [18]. Then, the samples were immersed in their respective solutions for 2 weeks, following which the final color and surface roughness measurements were recorded.

Color stability was assessed using an X-rite Ci7000 Series Benchtop Spherespectrophotometer (X-Rite, Inc., Grand Rapids, MI, USA) under D65 illumination against a 50% gray background. The color was measured in the CIE Lab* color space, where L* indicates lightness, a* indicates red–green balance, and b* indicates yellow–blue balance. The total color difference, ΔE, was calculated as previously described [19].

Surface roughness was analyzed with a Contour GT-K 3D Optical profilometer (Bruker Nano Surfaces, Tucson, AZ, USA) using Vertical Scan Interferometry at 5X magnification. Ra was calculated as the arithmetic average of the absolute values of peaks and valleys on the specimen’s surface [20,21,22].

Statistical analyses were performed separately for color change (ΔE) and surface roughness change (ΔRa), as these outcomes were evaluated using independent specimen sets. The color analysis included 10 specimens per material–solution combination (n = 10; total N = 120), whereas the surface roughness analysis included 5 specimens per material–solution combination (n = 5; total N = 60). For each color specimen, the differences between post-immersion and baseline CIELAB coordinates were calculated as ΔL*, Δa*, and Δb*. Total color difference was then calculated according to the CIE76 formula: ΔEab = [(ΔL)^2^ + (Δa*)^2^ + (Δb*)^2^]^1/2^. Surface roughness change was calculated for each specimen as ΔRa = Ra_post-immersion−Ra_baseline. Descriptive statistics were calculated for each material × solution combination and are reported as mean ± standard deviation (SD). For ΔE, assumptions were evaluated using residual diagnostics, the Shapiro–Wilk test, and Levene’s test. Residuals did not significantly deviate from normality (Shapiro–Wilk, *p* = 0.288), although Levene’s test indicated borderline heterogeneity of variance (*p* = 0.045). As the experimental design was balanced, a two-way factorial analysis of variance (ANOVA) was performed to evaluate the effects of material, immersion solution, and the material × solution interaction. Heteroscedasticity-robust inference was additionally examined as a sensitivity analysis, which yielded the same substantive conclusions. Following a significant interaction, simple-effects comparisons between materials within each immersion solution were performed using Welch’s *t*-tests with Holm adjustment for multiple comparisons. The partial eta squared (ηp^2^) was calculated as an effect size measure. For ΔRa, substantial non-normality and dispersion were observed in some experimental groups. The effects of material, immersion solution, and their interaction were therefore examined using the factorial model together with a permutation-based sensitivity analysis that preserved the factorial experimental structure. As solution demonstrated a significant overall effect, pairwise comparisons between immersion solutions were subsequently evaluated using Mann–Whitney tests with Holm adjustment for multiple comparisons.

No correlation analysis between ΔE and ΔRa was performed, as the color and surface roughness evaluations used independent specimen sets. Consequently, individual ΔE and ΔRa observations could not be validly paired for specimen-level correlation analysis. Statistical significance was established at α = 0.05, and multiplicity-adjusted *p*-values were used for post hoc comparisons.

## 3. Results

### 3.1. Color Analysis

A total of 120 specimens were evaluated for color change, comprising 10 specimens for each material × solution combination. ΔE was recalculated directly from the paired baseline and post-immersion CIELAB measurements using the CIE76 color difference formula.

#### 3.1.1. Descriptive Analysis

**Figure 1:**
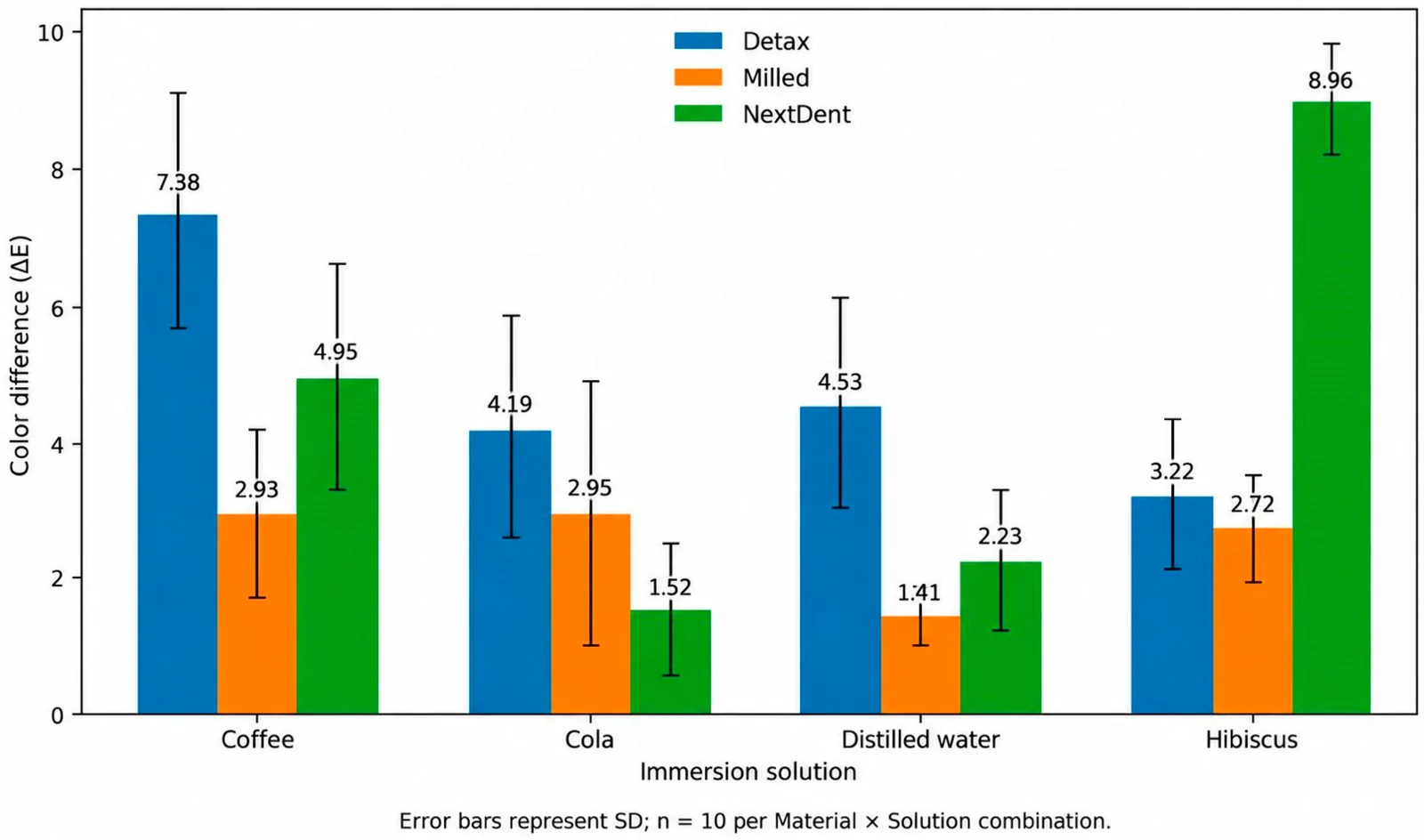
Mean color difference (ΔE) for the three provisional materials following exposure to the four immersion solutions. Error bars represent standard deviations (n = 10 per material × solution combination).

The color change values varied substantially according to the material and immersion solution (Table 1 and Figure 1). The highest mean ΔE was observed for NextDent immersed in hibiscus tea (8.96 ± 0.75), followed by Detax immersed in coffee (7.38 ± 1.69). In contrast, the lowest mean ΔE was observed for Vita CAD-Temp immersed in distilled water (1.41 ± 0.45), followed by NextDent immersed in cola (1.52 ± 1.00).

#### 3.1.2. Factorial Analysis of Color Change

The two-way factorial ANOVA results demonstrated the statistically significant effects of material type, immersion solution, and their interaction on ΔE (Table 2). Material had a significant overall effect on ΔE (F[2,108] = 35.65, *p* < 0.001, ηp^2^ = 0.398), as did immersion solution (F[3,108] = 28.64, *p* < 0.001, ηp^2^ = 0.443). Importantly, a significant material × solution interaction was also identified (F[6,108] = 29.36, *p* < 0.001, ηp^2^ = 0.620). The large interaction effect indicates that the influence of material type on color stability depended strongly on the immersion solution. Therefore, interpretation of the color findings was based primarily on simple-effects comparisons within individual solutions, rather than on the overall main effects alone.

#### 3.1.3. Simple-Effects Comparisons

As a significant material × solution interaction was observed, pairwise comparisons between materials were performed separately within each solution with Holm adjustment for multiple comparisons. Under coffee immersion, all three materials differed significantly. Detax demonstrated greater ΔE than Vita CAD-Temp (adjusted *p* < 0.001) and NextDent (adjusted *p* = 0.008), while NextDent demonstrated greater ΔE than Vita CAD-Temp (adjusted *p* = 0.008).

For cola, Detax demonstrated significantly greater ΔE than NextDent (adjusted *p* = 0.002), while the Detax versus Vita CAD-Temp (adjusted *p* = 0.145) and Vita CAD-Temp versus NextDent (adjusted *p* = 0.120) comparisons were not statistically significant. For distilled water, Detax demonstrated significantly greater ΔE than Vita CAD-Temp (adjusted *p* < 0.001) and NextDent (adjusted *p* = 0.002). Vita CAD-Temp and NextDent also differed significantly (adjusted *p* = 0.044). For hibiscus tea, NextDent demonstrated substantially greater ΔE than Detax and Vita CAD-Temp (both adjusted *p* < 0.001), whereas Detax and Vita CAD-Temp did not differ significantly (adjusted *p* = 0.270). These findings confirm a pronounced material-dependent response to the different immersion solutions.

### 3.2. Surface Roughness Change (ΔRa)

A total of 60 independent specimens were evaluated for surface roughness, comprising 5 specimens for each material × solution combination. The surface roughness change was calculated as post-immersion Ra minus baseline Ra.

#### 3.2.1. Descriptive Analysis

**Figure 2:**
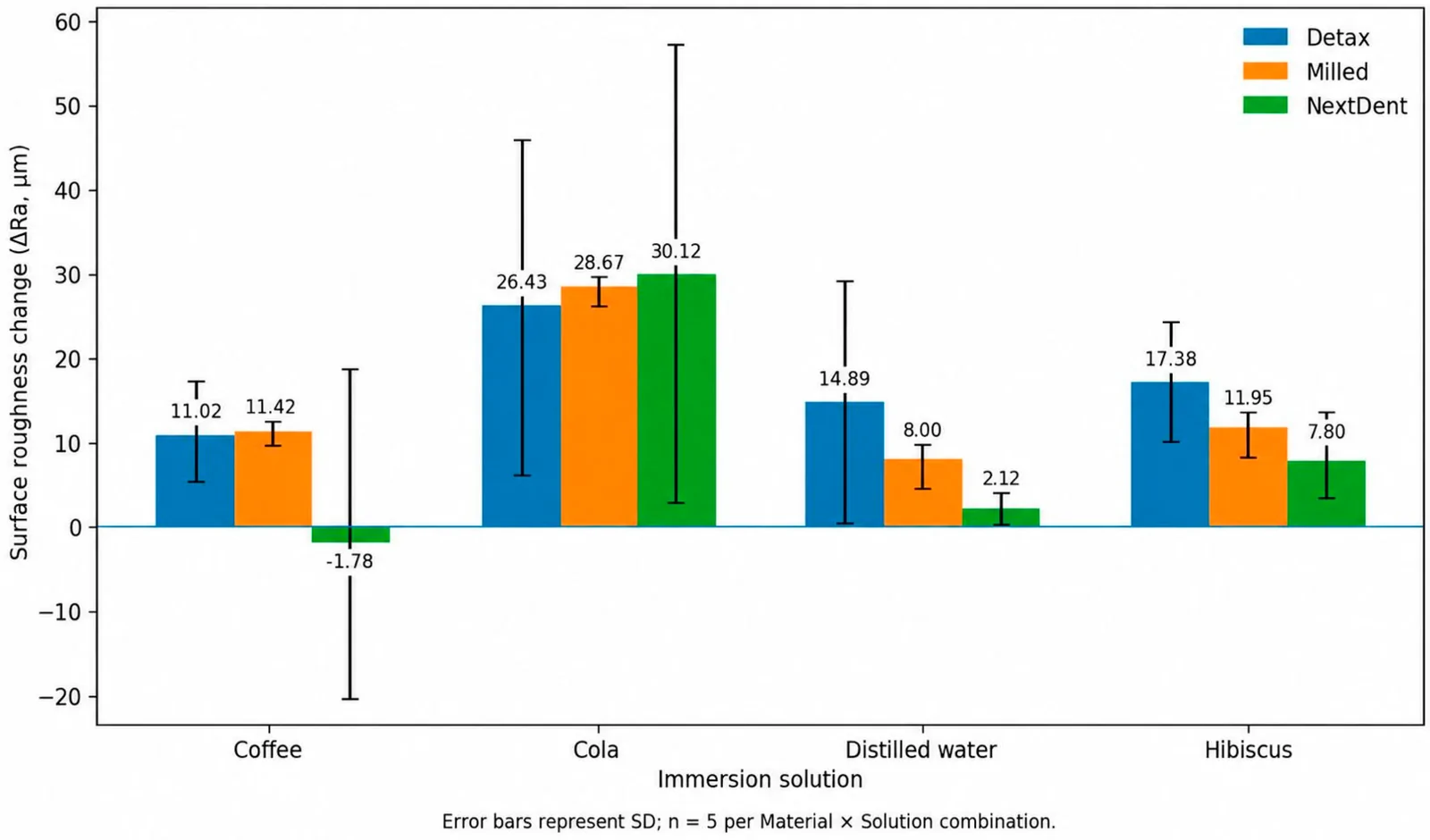
Mean change in surface roughness (ΔRa, µm) for the three provisional materials following exposure to the four immersion solutions. Error bars represent standard deviations (n = 5 per material × solution combination).

The descriptive results are presented in Table 3 and Figure 2. The greatest mean increase in surface roughness was observed for NextDent immersed in cola (30.12 ± 26.90 µm), followed by Vita CAD-Temp immersed in cola (28.67 ± 1.84 µm) and Detax immersed in cola (26.43 ± 19.72 µm). NextDent immersed in coffee demonstrated a mean ΔRa of −1.78 ± 20.56 µm. The large SDs reflect substantial specimen-to-specimen variability, including increases and decreases in surface roughness.

#### 3.2.2. Factorial Analysis of Surface Roughness

The factorial analysis showed a significant effect of immersion solution on ΔRa (F[3,48] = 9.39, *p* < 0.001), whereas the overall effect of material was not statistically significant (F[2,48] = 2.08, *p* = 0.136). Furthermore, no significant material × solution interaction was identified (F[6,48] = 0.63, *p* = 0.706) (Table 4). As residual normality was violated and considerable dispersion was present in several experimental groups, a permutation-based factorial sensitivity analysis was additionally performed. This analysis produced the same substantive interpretation: immersion solution significantly influenced ΔRa, whereas material type and the material × solution interaction did not show statistically significant effects.

#### 3.2.3. Comparisons Between Immersion Solutions

Given the significant overall effect of solution, post hoc comparisons were conducted between the immersion solutions with Holm adjustment. Across the materials, cola produced the greatest overall increase in surface roughness; in particular, ΔRa following cola exposure was significantly greater than that after coffee (adjusted *p* = 0.001), distilled water (adjusted *p* = 0.002), and hibiscus tea (adjusted *p* = 0.006). The difference between distilled water and hibiscus tea was also statistically significant (adjusted *p* = 0.048). Coffee did not differ significantly from distilled water (adjusted *p* = 0.124) or hibiscus tea (adjusted *p* = 0.361). Thus, the surface roughness findings primarily demonstrate an effect of immersion solution, rather than an overall effect attributable to material type.

## 4. Discussion

This study evaluated the effects of material type and immersion solution on the color stability and surface roughness of one CAD/CAM-milled and two 3D-printed provisional restorative materials following thermocycling and subsequent immersion in coffee, hibiscus tea, Coca-Cola, or distilled water. The findings demonstrated that color change was significantly influenced by material type, immersion solution and, importantly, the interaction between these two factors. In contrast, the change in surface roughness was significantly influenced only by immersion solution, as neither the overall effect of material type nor the material × solution interaction was statistically significant. Therefore, the null hypotheses related to the effects of material, solution, and their interaction were rejected for ΔE; whereas, for ΔRa, only the null hypothesis related to that of the immersion solution was rejected.

The significant material × solution interaction observed for ΔE is an important finding, as it demonstrates that color stability cannot be explained solely by whether a provisional material was milled or 3D-printed. Instead, the magnitude of discoloration depended on the specific combination of material and immersion solution. The highest mean color change was observed for NextDent following hibiscus tea exposure (ΔE = 8.96 ± 0.75), whereas Detax demonstrated the greatest color change following coffee exposure (ΔE = 7.38 ± 1.69). Conversely, NextDent demonstrated a comparatively low ΔE following cola exposure (1.52 ± 1.00). Vita CAD-Temp generally demonstrated relatively low ΔE values across the four solutions, ranging from 1.41 ± 0.45 in distilled water to 2.95 ± 1.96 in cola. These findings emphasize that the color behaviors of provisional materials are material- and environment-specific, rather than being determined by fabrication method alone.

Differences in color stability between provisional materials have previously been associated with several factors, including resin composition, polymerization characteristics, filler content, water sorption, surface characteristics, and the chemical composition of the staining medium [10,11,12,13,14,15,16,17,23,24,25,26]. Vita CAD-Temp was fabricated from industrially polymerized blocks under standardized manufacturing conditions, which may result in a relatively high degree of polymerization and reduced residual monomer content when compared with some additively manufactured resins. These characteristics may contribute to reduced water sorption and pigment penetration and may partly explain the relatively low ΔE values observed for Vita CAD-Temp in the present study [2,14,15]; however, the pronounced interaction effect observed in the present study indicates that generalizing this color performance to 3D-printed materials would be inappropriate. The two 3D-printed materials responded differently to the same immersion solutions; in particular, Detax demonstrated its greatest discoloration following coffee exposure, whereas NextDent demonstrated its greatest discoloration following hibiscus tea exposure. This suggests that differences in resin formulation and material-specific interactions with staining agents may be important determinants of discoloration. Previous investigations have similarly demonstrated that staining susceptibility varies between provisional restorative materials, depending on the restorative material and staining medium [10,11,12,14,16,17,27,28,29,30,31,32]. From a clinical perspective, the magnitude of the observed color differences is also relevant. Color difference thresholds are commonly used to distinguish changes that may be visually perceptible or clinically acceptable [25,26,29]. Several material × solution combinations in the present study demonstrated substantial ΔE values, particularly NextDent exposed to hibiscus tea and Detax exposed to coffee. Therefore, clinicians should consider not only the fabrication technique but also the expected duration of provisionalization and the patient’s exposure to potentially staining beverages when selecting a provisional restorative material. Nevertheless, any direct extrapolation of the presented in vitro ΔE values for the purpose of defining clinical acceptability should be made cautiously, as visual perception is influenced by several clinical variables that were not reproduced in the present experimental setting.

Printed provisional restorations have been shown to have structural porosity, which causes them to have increased surface roughness [5,6,12,14]. In contrast, CAD/CAM-milled materials better retain their mechanical properties even after prolonged exposure to acidic beverages. Due to their dense structure caused by pre-polymerization during fabrication, milled materials have better resistance to chemical and mechanical wear [2]. According to Tuncer et al. (2020), milled materials are more resistant to changes in roughness than 3D-printed and conventionally fabricated materials [4]. In addition, Lauvahutanon et al. (2017) highlighted that the compact structure of CAD/CAM materials provides enhanced protection against acidic erosion [2]. In the present study, coffee and distilled water (control) had lesser impacts on surface roughness when compared with Coca-Cola and hibiscus tea. Coffee’s less erosive effects were likely due to its lower acidity, although there were still noticeable changes in roughness. The surface roughness of dental materials is more strongly affected by immersion solutions with higher acidity, such as carbonated drinks, as has been reported by Mutlu-Sagesen et al. (2001) and Kotnarin et al. (2018) [5,6].

The results for surface roughness obtained in this study demonstrated a different pattern when compared with those for color stability. The immersion solution had a statistically significant effect on ΔRa, whereas material type and the material × solution interaction did not. Cola produced the greatest increase in surface roughness across the tested materials, with mean ΔRa values of 26.43 ± 19.72 µm for Detax, 28.67 ± 1.84 µm for the Vita CAD-Temp, and 30.12 ± 26.90 µm for NextDent. This finding suggests that, under the evaluated conditions, the immersion environment exerted a stronger overall influence on surface degradation than the fabrication method. The effect of cola on surface roughness may be associated with its acidic nature. Exposure of a material to acidic environments may contribute to degradation of its resin matrix, filler–matrix interface, or superficial polymer structure, thereby altering its surface topography [14,15]. Previous studies have reported that exposure to acidic beverages and staining media can affect the surface characteristics of resin-based restorative and provisional materials [5,6,12,13,14]. The present findings support the importance of the chemical environment in determining surface changes in provisional restorative materials.

Although numerical differences in ΔRa were observed between the three materials, the overall material effect was not statistically significant. Consequently, the present data do not support the conclusion that Vita CAD-Temp was statistically more resistant to a change in surface roughness than either of the 3D-printed materials. This distinction is important, because descriptive differences should not be interpreted as statistically demonstrated material superiority. Furthermore, substantial variability was observed in some groups—particularly NextDent exposed to coffee and cola. The NextDent–coffee group demonstrated a mean ΔRa of −1.78 µm with an SD of 20.56 µm, reflecting considerable inter-specimen variation. These findings warrant cautious interpretation, particularly because surface roughness was evaluated using five specimens per material × solution combination. Surface characteristics remain clinically relevant because increased roughness can influence plaque retention, bacterial adhesion, staining, and the interaction between restorative materials and surrounding periodontal tissues [20,21,22,31,32,33]. Nevertheless, the present results indicate that changes in surface roughness cannot be attributed solely to the fabrication technique. Under the evaluated experimental conditions, the immersion solution was the principal statistically significant factor affecting ΔRa.

Although surface roughness has been proposed as a factor that may influence susceptibility to staining, the present study did not directly evaluate a specimen-level association between ΔRa and ΔE, as the color and surface roughness measurements were made on independent specimen sets. Accordingly, no direct correlation between surface roughness change and color change can be inferred from the present data. The color differences observed between the materials are likely influenced by multiple material- and solution-dependent mechanisms rather than surface roughness alone.

The clinical implications of these findings are of note, as surface roughness and color stability are critical factors influencing the long-term success of provisional restorations. Increased surface roughness not only diminishes esthetics but also increases plaque accumulation, bacterial adhesion, and gingival inflammation [31,32,33]. Meanwhile, discoloration negatively impacts patient satisfaction and may necessitate the early replacement of restorations [3,4,7]. Given their superior resistance to surface roughness and color change, CAD/CAM-milled materials are an excellent option for provisional restorations, especially in esthetically demanding cases or for long-term use. However, 3D-printed materials—with their higher susceptibility to increased roughness and discoloration—may be better suited for short-term use (≤2 weeks). These materials could further benefit from additional surface treatments or coatings to improve their durability and esthetic performance, as suggested by Köroğlu et al. (2016) [8,34,35,36,37].

Although this study provides valuable insights, it has some limitations. The in vitro design may not fully replicate the complexities of the oral environment. Furthermore, this study evaluated a restricted number of materials, comprising only one milled and two 3D-printed provisional resins, thus precluding generalization of the study’s conclusions to milled versus 3D-printed materials as manufacturing categories. Furthermore, the differences observed between the two 3D-printed materials (NextDent and Detax) are likely attributable to differences in their organic matrix composition (monomer chemistry) rather than to the printing process itself. Future studies incorporating a larger number of commercially available milled and 3D-printed materials are needed to draw definitive, technique-level conclusions. Future studies should also focus on in vivo conditions to better understand the long-term performance of these materials [2,4,7]. Additionally, further research into the development of advanced 3D-printable materials with enhanced resistance to staining and surface degradation could expand their potential applications in the dental field.

## 5. Conclusions

Within the limitations of this in vitro study, color stability was significantly influenced by material type, immersion solution, and their interaction, demonstrating that the discoloration of provisional restorative materials depends on the specific material–solution combination. Vita CAD-Temp generally demonstrated relatively low color change values across the tested conditions, whereas the two 3D-printed materials showed more solution-dependent responses, with particularly high color changes observed for Detax following coffee exposure and NextDent following hibiscus tea exposure.

## Figures and Tables

**Table 1:** Color difference (ΔE) according to material and immersion solution.

Material	Coffee	Cola	Distilled Water	Hibiscus Tea
Detax	7.38 ± 1.69	4.19 ± 1.64	4.53 ± 1.52	3.22 ± 1.12
Vita CAD-Temp	2.93 ± 1.27	2.95 ± 1.96	1.41 ± 0.45	2.72 ± 0.81
NextDent	4.95 ± 1.62	1.52 ± 1.00	2.23 ± 1.06	8.96 ± 0.75

Values are mean ± SD; n = 10 per material × solution combination.

**Table 2:** Two-way factorial ANOVA results for ΔE.

Effect	df	F	*p*-Value	Partial η^2^
Material	2108	35.65	<0.001	0.398
Solution	3108	28.64	<0.001	0.443
Material × Solution	6108	29.36	<0.001	0.620

**Table 3:** Surface roughness change (ΔRa) according to material and immersion solution.

Material	Coffee	Cola	Distilled Water	Hibiscus Tea
Detax	11.02 ± 5.12	26.43 ± 19.72	14.89 ± 14.52	17.38 ± 7.33
Vita CAD-Temp	11.42 ± 1.28	28.67 ± 1.84	8.00 ± 3.74	11.95 ± 2.92
NextDent	−1.78 ± 20.56	30.12 ± 26.90	2.12 ± 1.71	7.80 ± 3.83

Values are mean ± SD in µm; n = 5 per material × solution combination.

**Table 4:** Factorial analysis results for ΔRa.

Effect	df	F	*p*-Value
Material	2,48	2.08	0.136
Solution	3,48	9.39	<0.001
Material × Solution	6,48	0.63	0.706

## Data Availability

The original contributions presented in this study are included in the article. Further inquiries can be directed to the corresponding author.
